# Reoperations are few and confined to the most valgus phenotypes 4 years after unrestricted calipered kinematically aligned TKA

**DOI:** 10.1007/s00167-021-06473-3

**Published:** 2021-02-13

**Authors:** Stephen M. Howell, Manpreet Gill, Trevor J. Shelton, Alexander J. Nedopil

**Affiliations:** 1 Department of Biomedical Engineering, University of California, Davis, 451 E. Health Sciences Drive, Room 2303, Davis, CA 95616 USA; 2 Adventist Health Lodi Memorial Hospital, 975 S. Fairmont Ave., Lodi, CA 95240 USA; 3 Department of Orthopaedic Surgery, Southern California Orthopedic Institute, 6815 Noble Ave., Van Nuys, CA 91405 USA; 4 Department of Orthopaedic Surgery, König-Ludwig-Haus, University of Würzburg, Würzburg, Germany

**Keywords:** Total knee arthroplasty, Total knee replacement, Kinematic alignment, Calipered, Reoperation, Phenotype

## Abstract

**Purpose:**

The present study determined the postoperative phenotypes after unrestricted calipered kinematically aligned (KA) total knee arthroplasty (TKA), whether any phenotypes were associated with reoperation, implant revision, and lower outcome scores at 4 years, and whether the proportion of TKAs within each phenotype was comparable to those of the nonarthritic contralateral limb.

**Methods:**

From 1117 consecutive primary TKAs treated by one surgeon with unrestricted calipered KA, an observer identified all patients (*N* = 198) that otherwise had normal paired femora and tibiae on a long-leg CT scanogram. In both legs, the distal femur–mechanical axis angle (FMA), proximal tibia–mechanical axis angle (TMA), and the hip–knee–ankle angle (HKA) were measured. Each alignment angle was assigned to one of Hirschmann’s five FMA, five TMA, and seven HKA phenotype categories.

**Results:**

Three TKAs (1.5%) underwent reoperation for anterior knee pain or patellofemoral instability in the subgroup of patients with the more valgus phenotypes. There were no implant revisions for component loosening, wear, or tibiofemoral instability. The median Forgotten Joint Score (FJS) was similar between phenotypes. The median Oxford Knee Score (OKS) was similar between the TMA and HKA phenotypes and greatest in the most varus FMA phenotype. The phenotype proportions after calipered KA TKA were comparable to the contralateral leg.

**Conclusion:**

Unrestricted calipered KA’s restoration of the wide range of phenotypes did not result in implant revision or poor FJS and OKS scores at a mean follow-up of 4 years. The few reoperated patients had a more valgus setting of the prosthetic trochlea than recommended for mechanical alignment. Designing a femoral component specifically for KA that restores patellofemoral kinematics with all phenotypes, especially the more valgus ones, is a strategy for reducing reoperation risk.

**Level of evidence:**

Therapeutic, Level III

## Introduction

The novel concept of categorizing the femoral mechanical angle (FMA), tibial mechanical angle (TMA), and hip–knee–ankle angle (HKA) into phenotypes by Hirschmann brought to light a wide variability in coronal alignment in the young nonosteoarthritic population, suggesting the need for a more individualized approach in total knee arthroplasty (TKA) [6–8]. The five FMA, five TMA, and seven HKA phenotypes each have a 3° range constructed from the mean value of the nonosteoarthritic knee. The phenotype with the mean value is considered ‘neutral’ alignment, which is different from mechanical alignment (MA) that considers components set perpendicular to the femoral and tibial's mechanical axes as neutrally aligned [6, 7]. The multiple phenotypes are a broader and more refined descriptor of the variability of alignment than the three categories of MA consisting of a ‘safe’-zone or neutral range (0 ± 3°), and the open-ended varus outlier (> 3° varus), and valgus outlier (> 3° valgus) ranges.

When performing TKA, the optimal postoperative coronal orientation of the femoral and tibial components and limb alignment remains unanswered [5]. Unrestricted calipered kinematic alignment (KA) is an individualized approach that strives to restore the patient’s prearthritic joints lines, limb, and *Q* angle or ‘phenotype’ without releasing healthy ligaments, including the posterior cruciate ligament [9]. Practitioners of calipered KA question the MA concept of right-angled femoral and tibial bone cuts to lines connecting the hip, knee, and ankle centers. These joint line orientations are rare and exist in only 0.1% of patients scheduled for TKA [1]. Consequently, MA changes the patient’s prearthritic femoral and tibial joint lines and *Q* angle in most patients, whereas calipered KA does not [6, 7]. However, unrestricted calipered KA can set the postoperative alignment outside the ‘safe’-zone recommended for MA [1].

The determination of whether unrestricted calipered KA TKA restores the patient’s prearthritic joint line and limb alignment is problematic because the radiographs show the preoperative deformity. A study of pairs of femora and tibiae without bone pathology showed the contralateral limb is a reasonable surrogate for coronal alignment since the side-to-side differences are small [3].

Since surgeons performing MA and restricted KA TKA are concerned about unrestricted calipered KA TKA restoring phenotypes outside their ‘safe’-zone, the present study determined the phenotypes in patients treated with unrestricted calipered KA TKA, which phenotypes were associated with a reoperation, implant revision, and lower outcome scores at a mean follow-up of 4 years, and whether the proportion of patients within each phenotype were comparable to the nonarthritic limb.

## Materials and methods

With approval from our institutional review board (IRB 1450380-1), an analysis of the senior author’s surgery schedule between September 2014 and 2017 identified 1117 consecutive primary TKAs treated with unrestricted calipered KA using cemented posterior cruciate retaining components and resurfacing of the patella. Each patient fulfilled the Centers for Medicare and Medicaid Services guidelines for medical necessity for TKA treatment. Included were osteoarthritic knee with (1) radiographic evidence of Kellgren–Lawrence Grade II to IV arthritic change or osteonecrosis; (2) any severity of clinical varus or valgus deformity); (3) and any severity of flexion contracture. On the day of discharge, each had an anteroposterior, rotationally controlled, nonweight-bearing, long-leg CT scanogram of both legs. These scans were reviewed by one author (TJS) independent of the treating surgeon and without knowledge of the reoperations, implant revision, and outcomes scores. The reviewer selected all patients (*N* = 198) with normal paired femora and tibiae other than the TKA for the study.

A single surgeon (SMH) performed the unrestricted calipered KA TKA through a mid-vastus approach and intraoperatively recorded a series of verification checks using a previously described technique [9]. For the femoral component, the I–E and varus–valgus (V–V) rotations and the A–P and proximal–distal (P–D) positions were set coincident with the native distal and posterior joint lines by adjusting the calipered thicknesses of the distal and posterior femoral resections to within 0 ± 0.5 mm of those of the femoral component condyles after compensating for cartilage wear and kerf of the saw blade. These steps reproducibly set the I–E rotation of the femoral component with a deviation of 0.3° ± 1.1° external from the F–E plane of the knee [19].

For the tibial component, the knee was balanced by adjusting the P–D position, V–V rotation, and the slope of the tibial resection according to six options in a decision tree. The V–V rotation of the resection was set coincident with the native proximal tibial joint line. The V–V angle of the tibial resection was adjusted, working in 1°–2° increments, until there was negligible medial and lateral liftoff of the trial insert from each condyle of the femoral component during a V–V laxity assessment in maximum extension with the spacer block and trial component. The proportion of patients with a proximal medial tibial angle of the tibial component within the normal left to right symmetry was 97% [21].

**Fig. 1:**
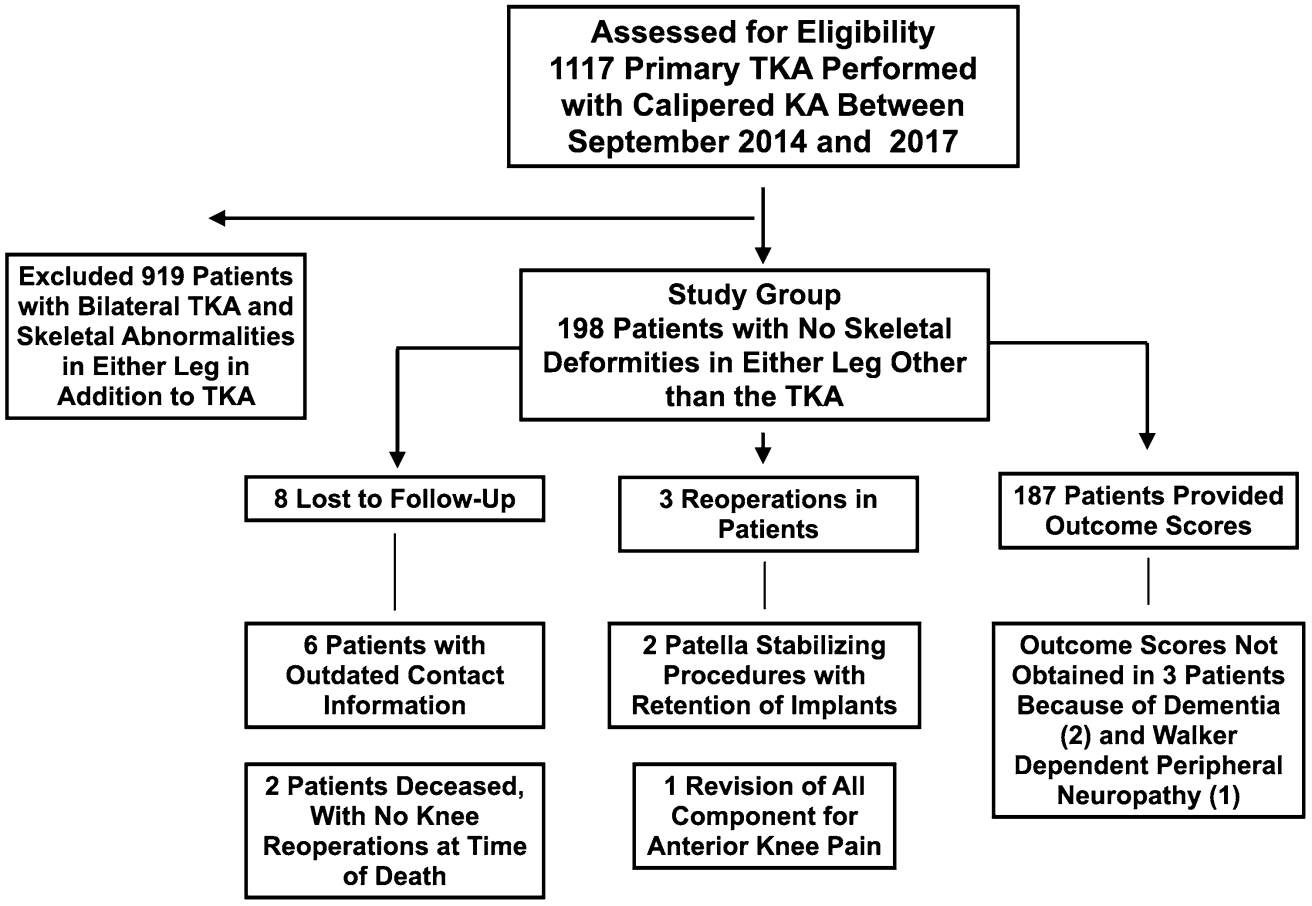
Flowchart shows the number of patients assessed for eligibility, excluded, included in the study group, lost-to follow-up, treated with reoperation, and the number that provided outcome scores

The number of patients assessed for eligibility, excluded, included in the study group, lost to follow-up, treated with reoperation, and the number with final follow-up outcome scores at a mean of 47 ± 8 months (range 33–66 months) is shown in Fig. 1. The average age of the 198 patients in the study was 67 ± 8 years, and 113 were females. The preoperative clinical characteristics are listed in Table 1.

**Table 1:** Preoperative clinical characteristics of included patients

Preoperative characteristics	Values (range)
Age	67 ± 8 years
Sex	113 females, 85 males
Body mass index	29 ± 5 kg/m^2^ (18– 43)
Extension	12° ± 7° (0–30)
Flexion	114° ± 7° (85–130)
Type of knee deformity	63% varus, 35% valgus, 2% patellofemoral
Radiographic knee deformity (+ Varus, − Valgus)	− 1° ± 7° (14 to − 17)
Kellgren–Lawrence classification	7% II, 47% III, 48% IV
Oxford score (48 is best, 0 is worst)	21 ± 8 points (3–35)
Knee society score (100 is best, 0 is worst)	32 ± 12 points (7–90)

A single observer (TJS) measured the FMA, TMA, and HKA on the limb with the TKA, using landmarks described by Hirschmann and free image analysis software (OsiriX Imaging Software, http://www.osirix-viewer.com) [7]. The contralateral limb was measured on a different day to minimize the risk of observation bias. The center of the femoral head was determined by best-fitting a circle. The center of the knee was the midline between the femoral condyles at the level of the distal joint line of the femoral component or native femur. The center of the ankle was the mid-width of the talus. The FMA was the lateral angle between the distal femoral joint line and the mechanical axis of the femur (varus > 90° and valgus < 90°). The TMA was the medial angle between the proximal tibial joint line and the mechanical axis of the tibia (varus < 90° and valgus > 90°) The HKA angle was the intersection of a line connecting the mechanical axes of the femur and tibia (varus was positive and valgus was negative). Each measurement was assigned to a phenotype category [6, 7].

Between September 2019 and March 2020, one observer (MKG) independent from the treating surgeon and blinded to the patient’s alignment contacted each patient by e-mail, postal service, and phone. Information from 5 “people search” websites updated outdated contact information. The patients were sent a questionnaire asking them whether they had a reoperation on the TKA and to complete and return the Forgotten Knee Score (100 best, 0 worst) and Oxford Knee Score (OKS) (48 best, 0 worst). The operative note identified the cause for surgery and the treatment provided for patients with a reoperation or implant revision.

### Statistical analysis

To quantify reproducibility, 3 observers independently performed the 3 radiographic measurements on both limbs on 20 randomly selected imaging studies. The intraclass correlation coefficient (ICC) and the 95% confidence interval (CI) were computed for each measurement with use of a 2-factor analysis of variance with random effects. The first factor was the observer with 3 levels (observers 1, 2, and 3). The second factor was the measurement of each of the 20 patients. An ICC value of > 0.9 indicates excellent agreement, and 0.75–0.90 indicates good agreement [12]. The ICC for the KA TKA and the contralateral limb was 0.93 (CI 0.82–0.97) and 0.95 (CI 0.87–0.98), respectively, for the HKA angle, 0.96 (CI 0.90–0.98) and 0.93 (CI 0.81–0.97), respectively, for the FMA, and 0.89 (CI 0.77–0.95) and 0.88 (CI 0.75–0.95), respectively, for the TMA, which indicates good to excellent agreement between the radiographic measurements made by 3 observers.

Discrete variables (patient-reported outcomes) were reported as number (percentage) (JMP Pro, 15.0.0, http://www.jmp.com). Continuous variables were reported as either the mean ± standard deviation (SD) or the median (interquartile range) depending on the normality of the data. A Wilcoxon/Kruskal–Wallis test determined the significance of the difference in the Forgotten Joint and Oxford Knee Scores between the FMA, TMA, and HKA phenotypes. Significance was *p* < 0.05. A Fisher’s Exact Test determined the significance of the difference in the proportions of the FMA, TMA, and HKA phenotypes between the calipered KA TKA and the contralateral, unaffected lower limb.

## Results

**Fig. 2:**
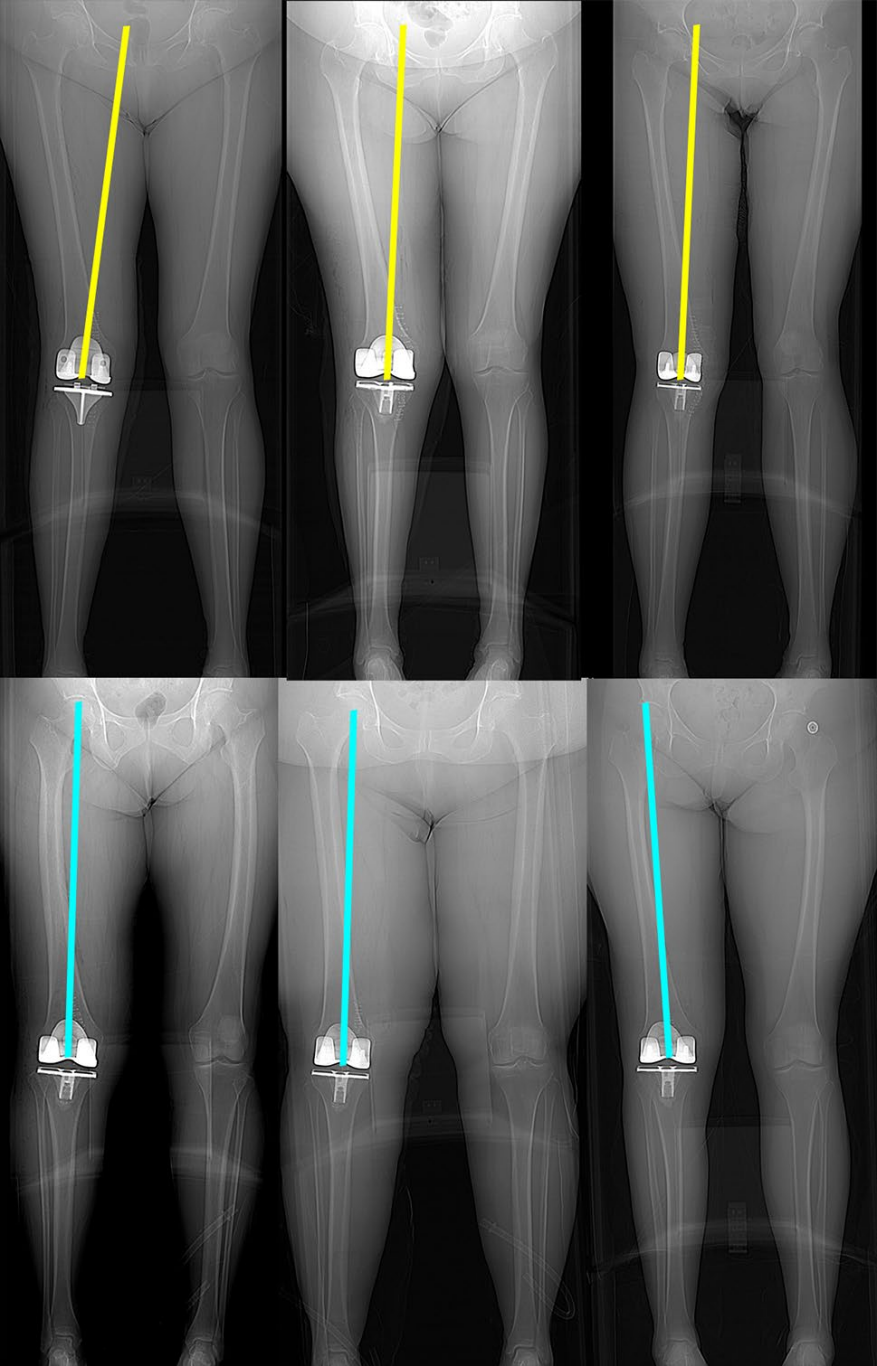
A composite of A–P scanograms shows the orientation of the prosthetic trochlea designed for MA for the three patients with reoperation (top row) projecting medially to the hip (yellow lines) compared to the projection to the center of the hip (blue line) of three randomly selected patients with neutral phenotypes (bottom row). For all six TKAs, the *Q* angle was comparable to the contralateral nonarthritic knee (not shown). The use of a prosthetic trochlea designed for MA and the kinematic alignment placement of the femoral component and prosthetic trochlea in a valgus orientation greater than the limit recommended for MA might explain the reoperations

**Fig. 3:**
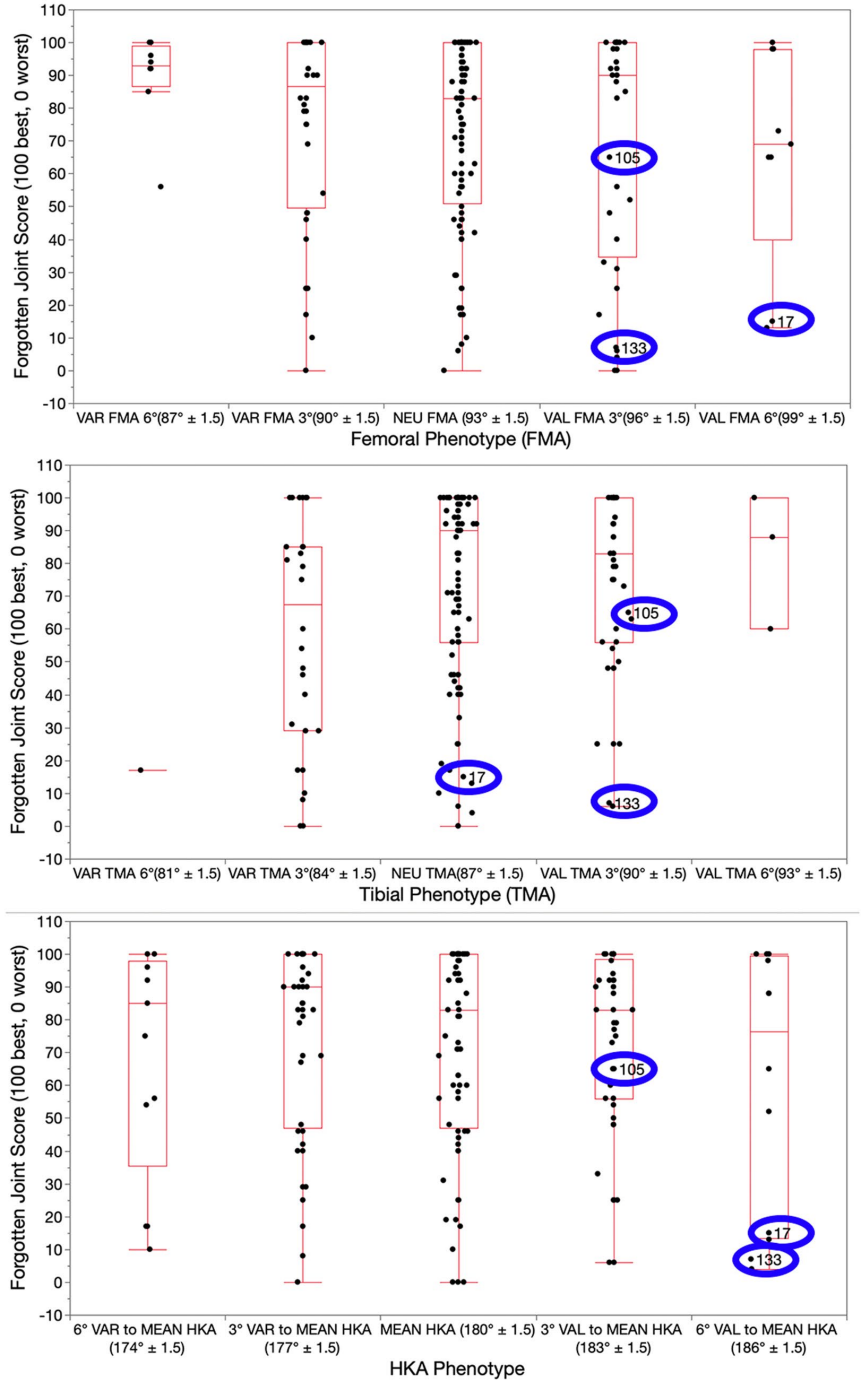
Box plots show that the median Forgotten Joint Score (transverse line within each red rectangle) 4 years after calipered KA TKA were not statistically different between the FMA, TMA, and HKA phenotypes, but trended lower for the most valgus FMA and HKA phenotype. The blue ovals identify the phenotypes and scores of those patients treated with a reoperation

**Fig. 4:**
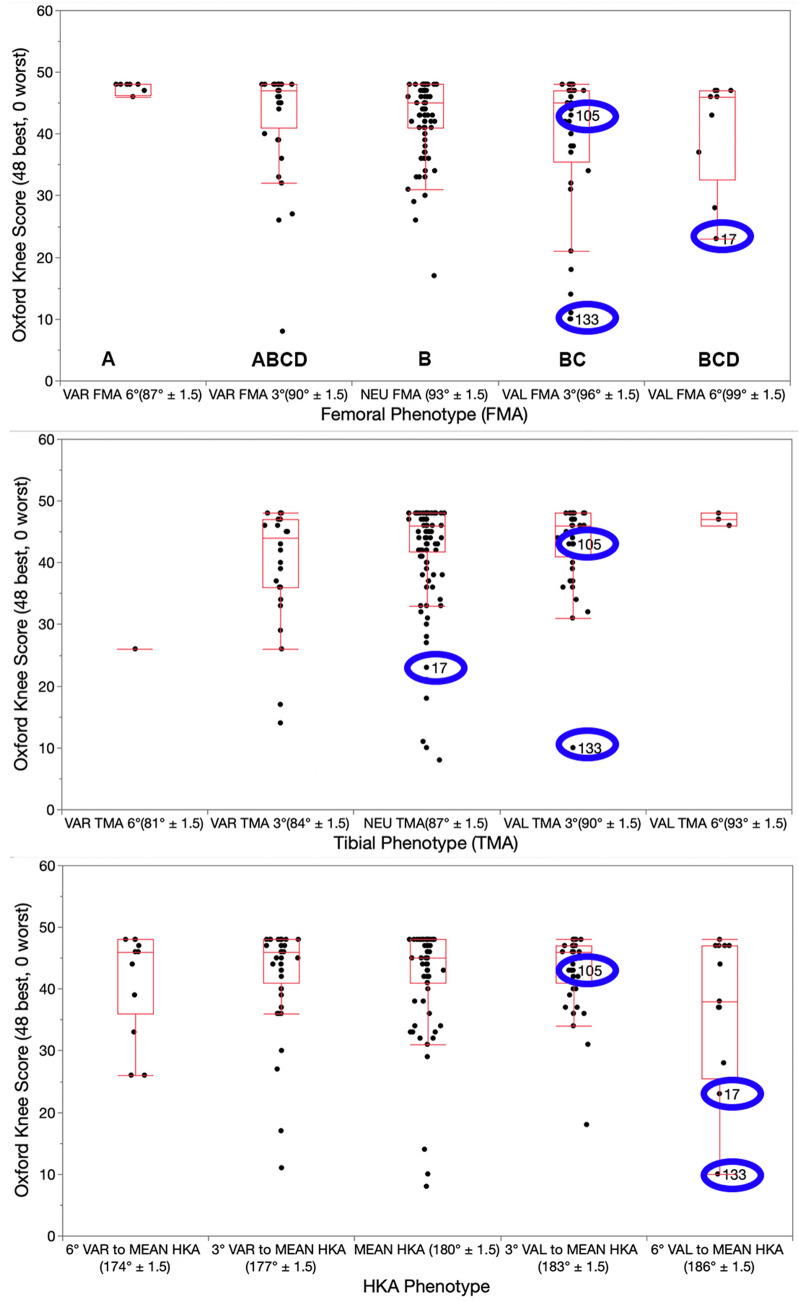
Box plots show the median Oxford Knee Score (transverse line within each red rectangle) after calipered KA TKA was not significantly different between the TMA and HKA phenotypes, but trended lower for the most valgus HKA phenotype. The median score of the most varus FMA phenotype was similar or greater than the other phenotypes (phenotypes with dissimilar letters are significantly different). The blue ovals identify the phenotypes and scores of those patients treated with a reoperation

**Fig. 5:**
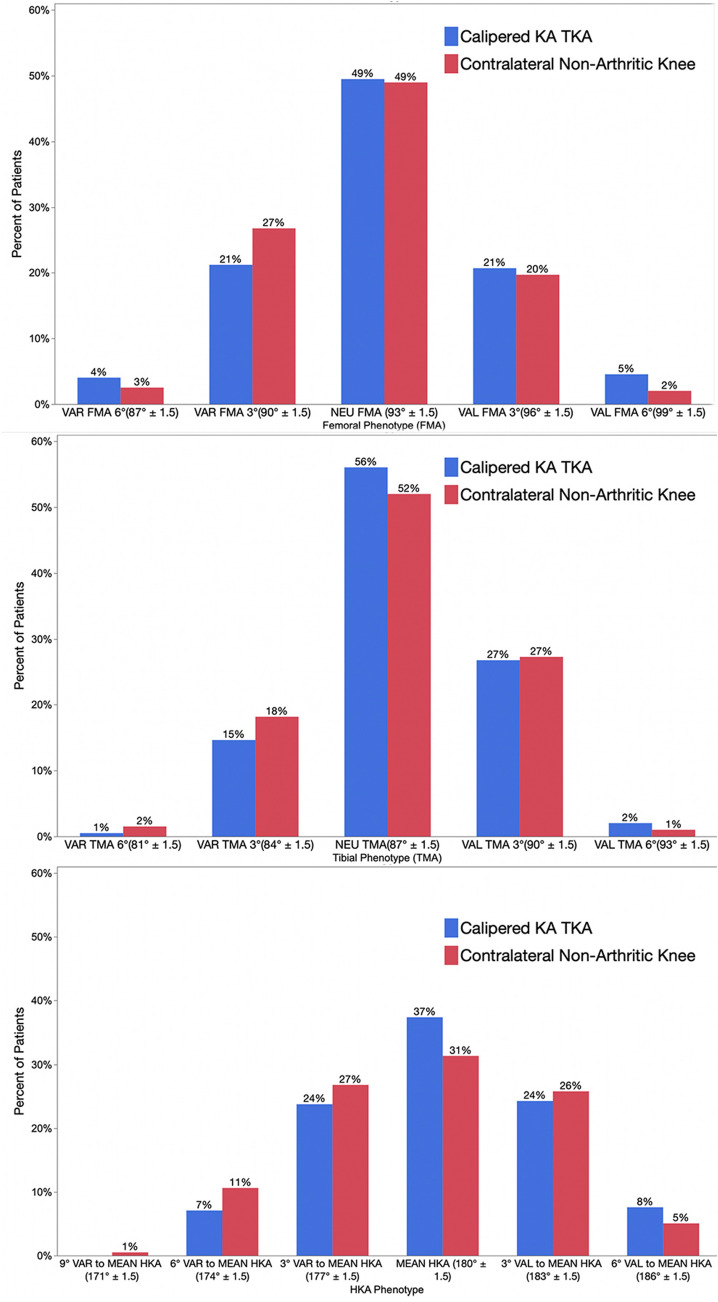
Column graphs show that the proportion of patients within each FMA, TMA, and HKA phenotype were comparable between the calipered KA TKA and the contralateral nonarthritic limb

The distribution of phenotypes in patients treated with unrestricted calipered KA TKA is displayed in Table 2. At a mean follow-up of 4 years, reoperation occurred in three females with the more extreme valgus phenotypes for a rate for all patients of 1.5% (3 of 198 (Fig. 2). One patient, with a valgus FMA 3°, valgus TMA 3°, and valgus HKA 6°, had an open lateral release and medial reefing with retention of components for patella subluxation by the senior author. Another had a valgus FMA 6°, neutral TMA, and valgus HKA 6° underwent an open lateral release and medial reefing with retention of components at another institution for anterior knee pain. The third, with a valgus FMA 3°, valgus TMA 3°, and valgus HKA 3°, underwent a full revision of components at another institution because of anterior knee pain. There were no revisions for component failure, loosening, or tibiofemoral instability. The Forgotten Joint Score was not statistically different between the FMA, TMA, and HKA phenotypes (*p* = 0.586, 0.971, and 0.858, respectively) (Fig. 3). The Oxford Knee Score was not significantly different between the TMA and HKA phenotypes (*p* = 0.221, and 0.295, respectively) (Fig. 4). The most varus FMA phenotype was associated with a greater Oxford Knee Score than three other FMA phenotypes (*p* = 0.029). The proportion of the FMA, TMA, and HKA phenotypes after calipered KA TKA was not different from those of the contralateral nonarthritic knee and limb (*p* = 0.427, 0.628, and 0.426, respectively) (Fig. 5 and Table 3).

**Table 2:** Absolute (*N*) and relative (%) distribution of limb (HKA), femoral (FMA), and tibial (TMA) phenotypes after calipered KA TKA

HKA			FMA			TMA		
Phenotypes	*N*	%	Phenotypes	*N*	%	Phenotypes	*N*	%
VAL_HKA_6°	15	7.6	VAL_FMA_6°	7	3.5	VAL_TMA_6°	4	2
VAL_HKA_3°	48	24.2	VAL_FMA_3°	43	21.7	VAL_TMA_3°	53	26.8
NEU_HKA_0°	74	37.4	NEU_FMA_0°	98	49.5	NEU_TMA_0°	111	56.1
VAR_HKA_3°	47	23.7	VAR_FMA_3°	42	21.2	VAR_TMA_3°	29	14.6
VAR_HKA_6°	14	7.1	VAR_FMA_6°	8	4.1	VAR_TMA_6°	1	0.5
Total	198	100	Total	198	100	Total	198	100

**Table 3:** Proportion of postoperative FMA, TMA, and HKA phenotypes matching the contralateral, unaffected leg

Phenotype	Same phenotype in KA TKA and contralateral leg (%)	KA TKA phenotype was 1 more varus than contralateral leg (%)	KA TKA phenotype was 1 more valgus than contralateral leg (%)
Femoral mechanical angle (FMA)	81	6	13
Tibial mechanical angle (TMA)	73	11	16
Hip–knee–ankle angle (HKA)*	70	9	19

*2% of calipered KA TKA had a HKA phenotype that was 2 more varus and 2 more valgus that contralateral leg

## Discussion

The most important findings of the present study were that (1) reoperations after unrestricted calipered KA TKA were for anterior knee pain and patellofemoral instability and were confined to those patients with the more valgus phenotypes, (2) implant revision for component wear, loosening, and tibiofemoral instability was negligible, (3) each phenotype had a comparable and high median FJS and OKS at a mean follow-up of 4 years, and (4) the phenotype proportions matched those of the paired nonarthritic limb.

The fact that the reoperations for patellofemoral symptoms were confined to three patients with the more valgus phenotypes is fortunate since it narrows the etiologies that foster this complication. One possible explanation is the valgus placement of the prosthetic trochlea outside the limit recommended for mechanical alignment (Fig. 2). The preferred varus–valgus MA orientation for the femoral component is within 0 ± 3° of the mechanical axis of the femur, which corresponds to the varus FMA 6° (87° ± 1.5°), varus FMA 3° (90° ± 1.5°), and neutral FMA (93° ± 1.5°) phenotype. All three patients with reoperation had a valgus FMA phenotype of either 3° or 6°, which is 6° and 9° more valgus than the MA varus–valgus target, respectively. One hypothetical solution is to use a femoral component designed explicitly for KA with a wider proximal trochlear to capture the patella.

The 1.3% incidence of reoperation for patellofemoral symptoms and 0% incidence of revision for implant failure or tibiofemoral instability at a mean follow-up of 4 years in the present study was comparable or lower than the 3% incidence of reoperation and revision for KA and MA reported by a registry study at a mean follow-up of 7 years [15]. One reason for the low incidence of reoperation for patellofemoral symptoms was the use of verification checks that reduce the risk of flexing the femoral component [4, 20]. A second is that KA restores patellofemoral kinematics and contact pressure distribution closer to the native knee than MA [14, 16]. A third is that KA of many commonly used femoral components restores the 3-dimensional native trochlea morphometry more closely than MA [11, 17, 24]. A fourth is that KA of the femoral component in internal rotation relative to the MA target cannot explain the reoperations' confinement to the small subgroup of TKAs with the more valgus phenotypes because the femoral component rotation was set coincident to the prearthritic posterior femoral joint line in all patients. The restoration of the patient's prearthritic *Q* angle explains the paradox that patellofemoral kinematics are better with KA, which is easily understood since MA increases and decreases the *Q* angle when treating knees with prearthritic constitutional varus and valgus limb alignment [22]. Hence, the reoperations for the patellofemoral symptom were more likely due to a more valgus orientation of the prosthetic trochlea than recommended for MA.

In the present study, there were no phenotypes with a clinically important lower median FJS and OKS. This finding is consistent with other studies of calipered KA TKA that showed no difference in patient-reported outcome scores between varus outlier range, valgus outlier range, and in-range according to MA criteria [10, 25]. Consequently, restoring the patient’s prearthritic phenotypes does not compromise patient-reported outcome scores.

The matching of the phenotype proportions with those of the contralateral limb in the present study is consistent with other studies that showed calipered KA achieves the target of restoring the patient’s prearthritic joint line [9, 21]. In the present study, ~ 85% of the FMA and TMA phenotypes had a side-to-side symmetry within ± one phenotype category (i.e., ± 3°). A series of 102 patients with a calipered KA TKA reported left to right symmetry within ± 3°, greater than 3° varus, and less than 3° valgus in 97%, 1%, and 2% for the FMA and 97%, 2%, and 1% for the TMA, respectively [21]. Hence, the calipered technique, verification checks, and fine tuning of the proximal tibial resection in increments of 1°–2° until the varus–valgus laxity of the knee in extension is negligible restores the patient’s prearthritic FMA, TMA, and HKA [13, 18, 21].

The following limitations affect the generalization of the results of the present study. A follow-up longer than 4 years could change the incidence and causes of reoperation. A study larger than 198 patients would increase the number of patient-reported outcomes scores in the most valgus and varus phenotypes and could reduce the risk of a Type II error from concluding these phenotypes did not have a lower FJS and OKS. The reoperation incidence could be related to the narrow and pointed geometry of the femoral component’s trochlea used in the present study. It might less for more robust trochlear designs and those explicitly developed for KA. Finally, these results are from a single-surgeon case series and a single component design. They should be confirmed by studies that assess multiple surgeons, different implant designs, and manual, patient-specific, navigation, and robotic instrumentation.

Surgeons using MA and restricted KA TKA might consider dropping the concept of a ‘safe’ zone for postoperative coronal component and limb alignment, which is too narrow according to multiple 10-year follow-up case-series of modern implant designs and materials that show revision for implant failure is no higher in the outlier ranges when compared with those in-range [2, 10, 23]. The present study showed that postoperative coronal alignment within the wide range of limb, femoral, and tibial phenotypes is within the ‘safe’ zone because of the lack of implant revision at a mean 4-year follow-up. This absence of implant revision is comparable to the 7-year findings of a registry study that compared KA to MA TKA, and a 10-year single-surgeon case series of unrestricted calipered KA [10, 15].

## Conclusion

A primary focus for those surgeons that perform unrestricted calipered KA TKA should be to lower the incidence of reoperation for anterior knee pain and patellofemoral instability confined to those patients with the more severe valgus phenotypes. Since the I–E rotation setting of the femoral component does not explain the reoperations, a promising strategy is to design a femoral component specifically for KA with a trochlea optimized in width, geometry, and orientation to capture the patella when used with any phenotype, especially the more valgus ones.
